# ^177^Lu-DOTA-HH1, a Novel Anti-CD37 Radio-Immunoconjugate: A Study of Toxicity in Nude Mice

**DOI:** 10.1371/journal.pone.0103070

**Published:** 2014-07-28

**Authors:** Ada H. V. Repetto-Llamazares, Roy H. Larsen, Anna Maria Giusti, Elena Riccardi, Øyvind S. Bruland, Pål Kristian Selbo, Jostein Dahle

**Affiliations:** 1 Nordic Nanovector AS, Oslo, Norway; 2 Department of Radiation Biology, Institute for Cancer Research, Oslo University Hospital, Montebello, Oslo, Norway; 3 Sciencons Ltd., Oslo, Norway; 4 Accelera Srl., Nerviano (Milano), Italy; 5 Department of Oncology, Norwegian Radium Hospital, Oslo University Hospital, Oslo, Norway; 6 Institute for Clinical Medicine, University of Oslo, Oslo, Norway; University Health Network, Canada

## Abstract

**Background:**

CD37 is an internalizing B-cell antigen expressed on Non-Hodgkin lymphoma (NHL) and chronic lymphocytic leukemia cells (CLL). The anti-CD37 monoclonal antibody HH1 was conjugated to the bifunctional chelator p-SCN-Bn-DOTA and labelled with the beta-particle emitting radionuclide ^177^Lu creating the radio-immunoconjugate (RIC) ^177^Lu-DOTA-HH1 (^177^Lu-HH1, trade name Betalutin). The present toxicity study was performed prior to initiation of clinical studieswith ^177^Lu-HH1.

**Methodology/Principal Findings:**

Nude mice with or without tumor xenografts were treated with 50 to 1000 MBq/kg ^177^Lu- HH1 and followed for clinical signs of toxicity up to ten months. Acute, life threatening bone marrow toxicity was observed in animals receiving 800 and 1000 MBq/kg ^177^Lu-HH1. Significant changes in serum concentrations of liver enzymes were evident for treatment with 1000 MBq/kg ^177^Lu-HH1. Lymphoid depletion, liver necrosis and atrophy, and interstitial cell hyperplasia of the ovaries were also observed for mice in this dose group.

**Conclusions/Significance:**

^177^Lu-DOTA-HH1 was well tolerated at dosages about 10 times above those considered relevant for radioimmunotherapy in patients with B-cell derived malignancies.The toxicity profile was as expected for RICs. Our experimental results have paved the way for clinical evaluation of ^177^Lu-HH1 in NHL patients.

## Introduction

NHL patients are conventionally treated with the anti-CD20 antibody rituximab alone or in combination with chemotherapy. After relapse only a fraction of the patients will be treated with the clinically approved anti-CD20 RICs Bexxar or Zevalin. However, a plausible novel approach could be to target a different antigen than CD20 at this stage of the disease. The CD37 antigen is a member of the tetraspanin transmembrane family and is expressed in B-cells from pre-B to peripheral mature B-cells, but is absent on plasma cells and normal stem cells [1]. CD37 internalizes, but has modest shedding in transformed B-cells expressing this antigen [2], [3]. Therefore, CD37 seems to be an appropriate therapeutic target in patients with relapsed B-cell derived malignancies, such as B-cell CLL, hairy-cell leukemia (HCL) and B-cell NHL.

Radio-immunotherapy (RIT) with CD37 as the target has previously been explored using a ^131^I-labeled murine monoclonal antibody (MB-1) both in a mouse model and in patients [4]–[9]. A higher degree of internalization and degradation of ^131^I-labeled RIC was found for CD37 than for CD20 [9]. Despite promising clinical responses observed in these clinical studies, further development of RIT focused on CD20 as the target antigen. To our knowledge, no subsequent efforts have been made to develop RIT with anti-CD37-based RICs.

Iodine-131 labeled via chloramine-T is a non-residualizing radionuclide which may be sub-optimal when targeting an internalizing antigen [10]. A switch to a residualizing radionuclide like ^177^Lu, labeled through a DOTA linker, may improve the properties of CD37 directed RIT. The metallic beta-emitter ^177^Lu (T_1/2_ = 6.7 days) has been successfully used in several clinical trials [11]–[15]. It is produced by direct neutron activation of ^176^Lu, or via beta decay of reactor-produced ^177^Yb and it is commercially available in GMP quality [16], [17]. ^177^Lu-based RIT seems appropriate in NHL where the stroma is less compact than in solid cancers allowing better diffusion of the RIC. The energy of the beta particle of ^177^Lu is relatively low, resulting in a shorter range in tissues compared to other beta-emitters used for RIT [17].

In an effort to re-evaluate and improve RIT against CD37 we have developed a new RIC (Betalutin) based on ^177^Lu linked to the anti-CD37 antibody HH1 (HH1), originally developed at the Norwegian Radium Hospital [18], via the backbone substituted chelator p-SCN-Bn-DOTA (DOTA or tetraxetan).

Severe Combined Immunodeficiency (SCID) mice, intravenously injected with Daudi lymphoma cells that developed tumors in the spine, lymph nodes, kidneys and lungs were successfully treated with ^177^Lu-HH1 [19]. The median survival of mice treated with 50 MBq/kg ^177^Lu-DOTA-HH1 increased by 55 days compared to untreated control mice. The maximum tolerated dosage in this radiosensitive strain of mice [20] was between 50 and 100 MBq/kg. A dosage of 50 MBq/kg or 100 MBq/kg equals an absorbed radiation dose between 2.9 and 5.8 Gy to tumor [21]. However, higher absorbed radiation doses will most probably be necessary for curative treatment of macroscopic tumors. It is therefore mandatory to study the toxicity of ^177^Lu-HH1 in a mouse strain that has intact DNA-damage-repair capability, such as conventional nude mice, where higher doses can be given and relevant therapeutic effects may be obtained. Although tumor models based on SCID mice might be interesting tools [22], their radiation sensitivity might lead to results that are more distant from reality than more conventional models.

The current paper evaluates the toxicity of ^177^Lu-HH1 in nude mice. Studying systemic toxicity of this newly developed RIC will guide in establishing a safe starting dosage for clinical trials and may give an indication of the potential side effects to be monitored during Phase I clinical trials. Dosages up to 1000 MBq/kg, corresponding to absorbed radiation doses to tumor of up to 58 Gy [21], were evaluated in the current study in mice, by measurements of survival (up to 10 months), body weight, hematology and histopathology.

## Materials and Methods

### Labeling of Antibodies with ^177^Lu

The chelator p-SCN-Bn-DOTA (DOTA) was dissolved in 0.005 M HCl, added to the antibody in a 6∶1 ratio and pH-adjusted to approximately 8.5 using a carbonate buffer. After 45 minutes of incubation at 37°C the reaction was stopped by the addition of 50 µl of 0.2 M glycine solution per mg of Ab. To remove free DOTA the conjugated antibody was washed using AMICON-30 centrifuge tubes (Millipore, Cork, Ireland) 4–5 times with NaCl 0.9%. Before labeling with ^177^Lu the pH was adjusted to 5.3±0.3 using 0.25 M ammonium acetate buffer. Between 120 and 220 MBq of ^177^Lu (Perkin Elmer, Boston, Ma, USA) were added to 1 mg of DOTA-Ab, and incubated for 45 minutes at 37°C. The radiochemical purity (RCP) of the conjugate was evaluated using instant thin layer chromatography. If RCP was below 95% the conjugate was purified using Econo-Pac 10 DG columns (Bio-rad Laboratories, California, USA)

### Immunoreactivity

Single cell suspensions of Daudi or Ramos lymphoma cells (both from ATCC in collaboration with LGC Standards, Boras, Sweden)) were grown in RPMI 1640 medium (PAA, Linz, Austria) supplemented with 10% heat-inactivated FCS (PAA), 1% L-glutamine (PAA) and 1% penicillin-streptomycin (PAA) in a humid atmosphere with 95% air/5% CO_2_. The immunoreactivity of the RIC was measured using a modified Lindmo method [23]. Cell concentrations of 10, 50, 100 and 200 million cells/ml were used in the tests. The immunoreactivity of all RICs used in the experiments was between 63% and 80%.

### Animals

Institutionally bred female athymic nude mice were used in the experiments (Table 1). Balb/C nu/nu (NCR) mice were used in experiments 1 and 2 (long term toxicity without tumor xenografts) while athymic nude Foxn1^nu^ were used in experiments 3 and 4 (Ramos xenografts). The animals were maintained under pathogen-free conditions with a 12 hours lighting cycle at a room temperature of 23°C and air relative humidity of 55% in plastic cages with 4 to 10 mice per cage. Food and water were supplied ad libitum and bedding was changed regularly. For xenograft implantation, mice were anesthetized with subcutaneous injections of 70–100 µl Tiletamin-Zolazepam mix (Virbac, Carros Cedex, France), diluted 1∶5 with sterile water before implantation of pieces of tumor tissue with diameters of about 1.5–2 mm. Treatments were administered by tail vein injection of around 100 µl solution to each mouse (adjusted to individual body weight). All procedures and experiments involving animals in this study were approved by the Norwegian Animal Research Authority and carried out according to the European Convention for the Protection of Vertebrates Used for Scientific Purposes. The procedures were approved by the Chief Veterinarian, Lena Kjempengren, at the Department of Comparative Medicine, Norwegian Radium Hospital.

**Table 1:** Overview of experiments.

Exp #	Groups	Number of mice	Follow-up time (weeks)	Mouse age (weeks)	Mouse weight (g)
**1**	NaCl, 50 and 100 MBq/kg ^177^Lu-HH1	8	40	16	17–22.2
**2**	NaCl, 150 and 250 MBq/kg ^177^Lu-HH1	10	24–40	16	24.1–35.1
**3**	NaCl, rituximab, HH1, 50, 100, 200 and 400 MBq/kg ^177^Lu-HH1	4–5	6	7–8	21.2–28.9
**4**	NaCl, 400, 800 and 1000 MBq/kg ^177^Lu-HH1	9–10	10	7–8	18.5–24.5

### Treatments

Four different experiments were performed (Table 1). One cage per treatment group was used. Mice were allocated in the cages so that the tumor size distribution was similar in each group. In experiment 1, 8 mice in each group were treated with a single administration of 0.9% NaCl, 50 or 100 MBq/kg ^177^Lu-HH1 and followed for 10 months. The mice in this experiment were initially implanted with Daudi tumors, but the take was low and the mice in which the tumors did not grow were selected for the experiment. These mice were considered to be without tumor. In experiment 2, groups of 10 mice without tumor xenografts were treated with a single administration of either 0.9% NaCl, 150 or 250 MBq/kg ^177^Lu-HH1. Half of the mice were monitored for 6 months and half for 10 months post-injection. In experiment 3, nude mice with Ramos lymphoma xenografts were treated with a single administration of 0.9% NaCl, unlabeled HH1, rituximab, or 50, 100, 200 or 400 MBq/kg ^177^Lu-HH1. Mice were followed for 4 to 6 weeks. In experiment 4, 9 or 10 mice with Ramos lymphoma xenografts were treated with a single administration of 0.9% NaCl, or 400, 800 or 1000 MBq/kg of ^177^Lu-HH1, and followed for 10 weeks. The end points studied in these experiments were weight loss higher than 20% (from baseline or highest recorded weight) and/or any sign of illness or discomfort. Mice were euthanized by neck dislocation whenever an end point was reached or when the experiment was finalized.

### Measurements

#### Body weight and tumor volume

The mice were weighed and evaluated clinically three times a week. Tumor size was measured 2 to 3 times a week using an electronic caliper in 2 dimensions. Tumor volume was calculated assuming an elliptical shape using the equation (a^2^×b/2) where *a* is the shortest and b the longest of the perpendicular axes.

#### Hematology

For blood cell counts, 50–75 µl blood was collected from the vena saphena lateralis in 0.5 ml EDTA coated tubes (BD Microtainer K2E tubes, Becton, Dickinson and Company, Franklin Lakes, NJ). Blood cells were counted using an automated hematology analyzer (Scil Vet abc animal blood counter, ABX Diagnostics, Montpellier Cedex 04, France). White blood cells (WBC), red blood cells (RBC) and platelets (PLT) were monitored.

#### Clinical Chemistry

For measurement of clinical chemistry parameters, 100–150 µl blood from the vena saphena lateralis was collected in heparin coated tubes (BD Microtainer K2E tubes, Becton, Dickinson and Company, Franklin Lakes, NJ). Alkaline phosphatase (ALP), Aspartate transaminase (GOT), Alanine transaminase (GPT) and Urea were measured in a Reflovet Plus (Roche Diagnostics, GmbH, D-68298, Mannheim, Germany). In some mice from experiment 4, blood samples were taken by heart puncture under gas anesthesia (Sevofluorane, Abbot, Abbot Park, IL).

### Statistics

All statistical analyses were performed using the software SigmaPlot 12.0 (Systat Software Inc, San Jose, California, USA). Survival curves were analyzed using Kaplan-Meier Survival Analysis and the Log-Rank test. Where multiple comparisons were required, the Holm-Sidak method for all multiple pairwise comparisons was used. Statistical analysis of the hematology and clinical chemistry data was performed using Student t-test with 0.05 as the threshold for significance.

### Histopathology

Organs/tissues were either fixed in formalin or in formalin free fixative (Accustain, Sigma Aldrich Co., St. Louis, USA,), processed for histological examination and embedded in paraffin wax. Histological sections were stained with hematoxylin and eosine and examined microscopically. The following organs/tissues were collected for histology examination from experiments 1, 2 and 3: stomach, duodenum, jejunum, ileum, cecum, colon, rectum, pancreas, mesenteric lymph nodes, aorta, lungs, eyes, lacrimal glands, thyroid/parathyroid, adrenals, ovaries, urinary bladder, kidneys, liver, gall bladder, vagina, uterus, sciatic nerve, skeletal muscle, cerebrum, cerebellum, esophagus, trachea, salivary glands, skin, femur, heart, spleen, muscle, lymph nodes/skin, and fatty tissue in the first two long term experiments. In experiment 4 fewer organs were sampled: heart, lungs, liver, stomach, spleen, small intestine, large intestine, kidneys, femur, muscle, thyroid, skull, brain, tumor, urinary bladder skin, ovaries and lymph nodes.

## Results

### Survival

**Figure 1:**
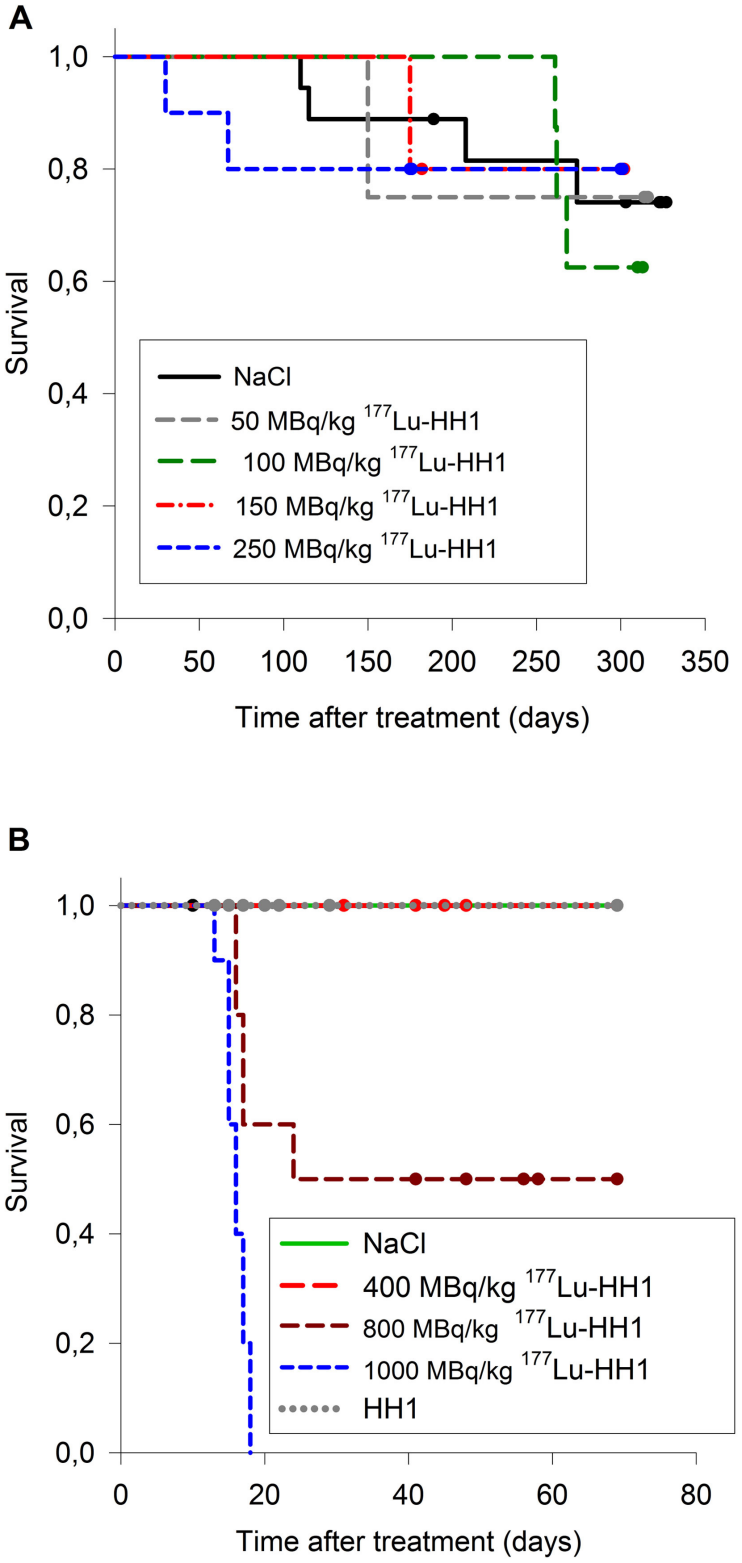
Survival of mice treated with increasing dosages of ^177^Lu-HH1. **A**. Mice without xenografts treated with saline or 50, 100, 150 or 250 MBq/kg ^177^Lu-HH1 (experiments 1 and 2). The end point for survival was euthanasia due to weight loss or substantial discomfort. **B**. Mice with Ramos xenografts treated with saline or 400, 800 or 1000 MBq/kg ^177^Lu-HH1 (experiment 4). The end point for survival was euthanasia due to WBC<1.5 10^9^ cells/L (usually accompanied by RBC<5 10^12^ and PLT<400 10^9^ cells/L), weight loss higher than 15% from base line, and/or substantial discomfort, all symptoms associated with radiation toxicity. Mice euthanized because of tumor diameter larger than 20 mm were censored.

The mice in experiments 1 and 2 were treated with saline or 50–250 MBq/kg ^177^Lu-HH1 (Table 1). The mice were euthanized if the weight loss was greater than 20% relative to the maximum measured weight, and/or if they appeared to experience substantial discomfort (Figure 1 A). There were no statistically significant differences in survival of mice treated with 50–250 MBq/kg ^177^Lu-HH1 and saline (p>0.985, All Pairwise Multiple Comparison Procedures, Holm-Sidak method). None of these mice were euthanized due to suspected radiation toxicity (no symptoms of sickness or discomfort or WBC lower than 1.5×10^9^ cells/L, RBC lower than 5×10^12^ cells/L and/or PLT lower than 400×10^9^ cells/L,), indicating that 250 MBq/kg and below appeared to be safe.

Mice in experiments 3 and 4 were treated with saline, cold rituximab, cold HH1, or 50–1000 MBq/kg ^177^Lu-HH1 (Table 1). The mice were euthanized if the WBC was lower than 1.5×10^9^ cells/L, the RBC was lower than 5×10^12^ cells/L and the PLT was lower than 400×10^9^ cells/L, and/or the weight loss was greater than 15% from highest recorded weight, and/or they appeared to experience substantial discomfort, which was considered to be an indication of radiation toxicity (Figure 1 B). In experiment 3, there were no statistical differences in survival between any of the groups (data not shown). Similarly, in experiment 4 the survival of the mice treated with 400 MBq/kg ^177^Lu-HH1 treatment was not significantly different from the survival of the control mice. On the other hand, half of the mice treated with 800 MBq/kg and all of the mice treated with 1000 MBq/kg showed symptoms of radiation toxicity and were euthanized between 13 and 17 days after injection, with one mouse treated with 800 MBq/kg ^177^Lu-HH1 being euthanized 24 days after start of injection due to symptoms of radiation toxicity. The survival of the mice treated with 1000 MBq/kg was significantly lower than that observed for all other treatments (p<0.002). The survival of the mice treated with 800 MBq/kg ^177^Lu-HH1 was significantly lower than for mice treated with HH1 or 400 MBq/kg ^177^Lu-HH1 (p<0.04), but not for mice treated with NaCl (p = 0.053). The reason for this result was that the mice in the control group were euthanized due to tumor size and therefore censored out of the survival analysis. The results for tumor growth will be reported elsewhere.

### Hematology

**Figure 2:**
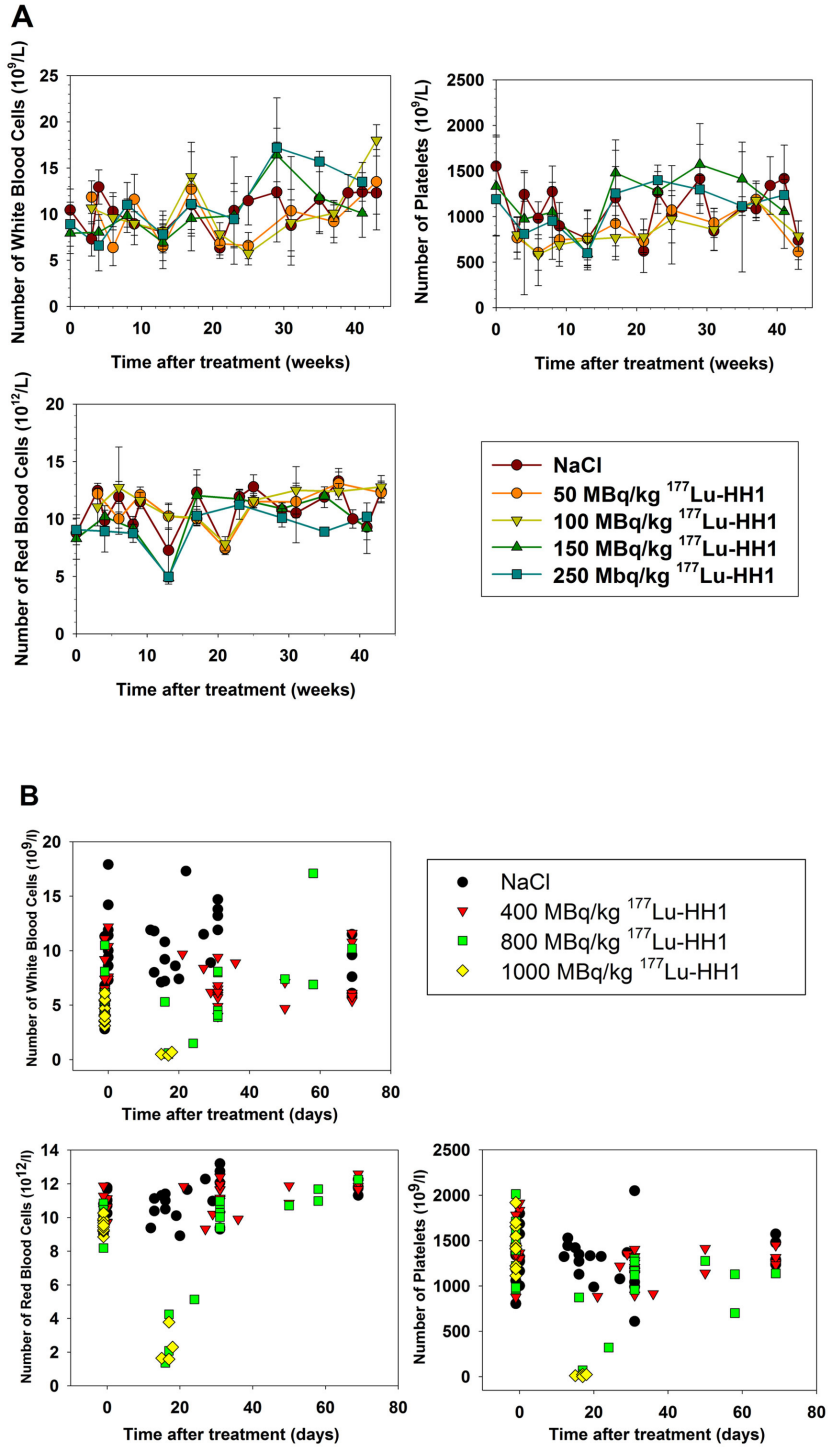
Effects of increasing dosage of ^177^Lu-HH1 on the number of white blood cells, red blood cells and platelets. **A**. Mice without xenografts treated with saline or 50, 100, 150 or 250 MBq/kg ^177^Lu-HH1 (experiments 1 and 2). Blood samples were taken every 3 to 4 weeks, N = 4–10. Error bars  =  Standard deviation. **B**. Mice with Ramos xenografts treated with saline or 400, 800 or 1000 MBq/kg ^177^Lu-HH1 (experiment 3 and 4). Blood samples were taken before treatment, 1 month after treatment and at euthanasia.

WBC, RBC and platelets were measured before injection and at several time points after injection in experiments 1 and 2, and before injection and at euthanasia in experiments 3 and 4. Blood counts in mice without xenografts treated with up to 250 MBq/kg of ^177^Lu-HH1 were similar to those of the control groups (Figure 2A). Blood counts in mice with Ramos xenografts treated with doses from 50 to 400 MBq/kg of ^177^Lu-HH1 and with cold antibodies were not significantly different from NaCl controls (Figure 2B, data from 50 to 200 MBq/kg of ^177^Lu-HH1 and cold antibodies not shown). In contrast, the blood counts at euthanasia for mice treated with 800 and 1000 MBq/kg ^177^Lu-HH1 were significantly lower than for mice treated with NaCl and HH1 (p<0.05) (Figure 2 B). For mice treated with 800 MBq/kg ^177^Lu-HH1 compared to mice treated with NaCl, the difference in the WBC was not statistically significant (p = 0.085), which is related to the fact that half of the mice treated with 800 MBq/kg ^177^Lu-HH1 survived the initial radiotoxicity and thereby their WBC had time to recover before they were measured (Figure 2 B).

### Body Weight

**Figure 3:**
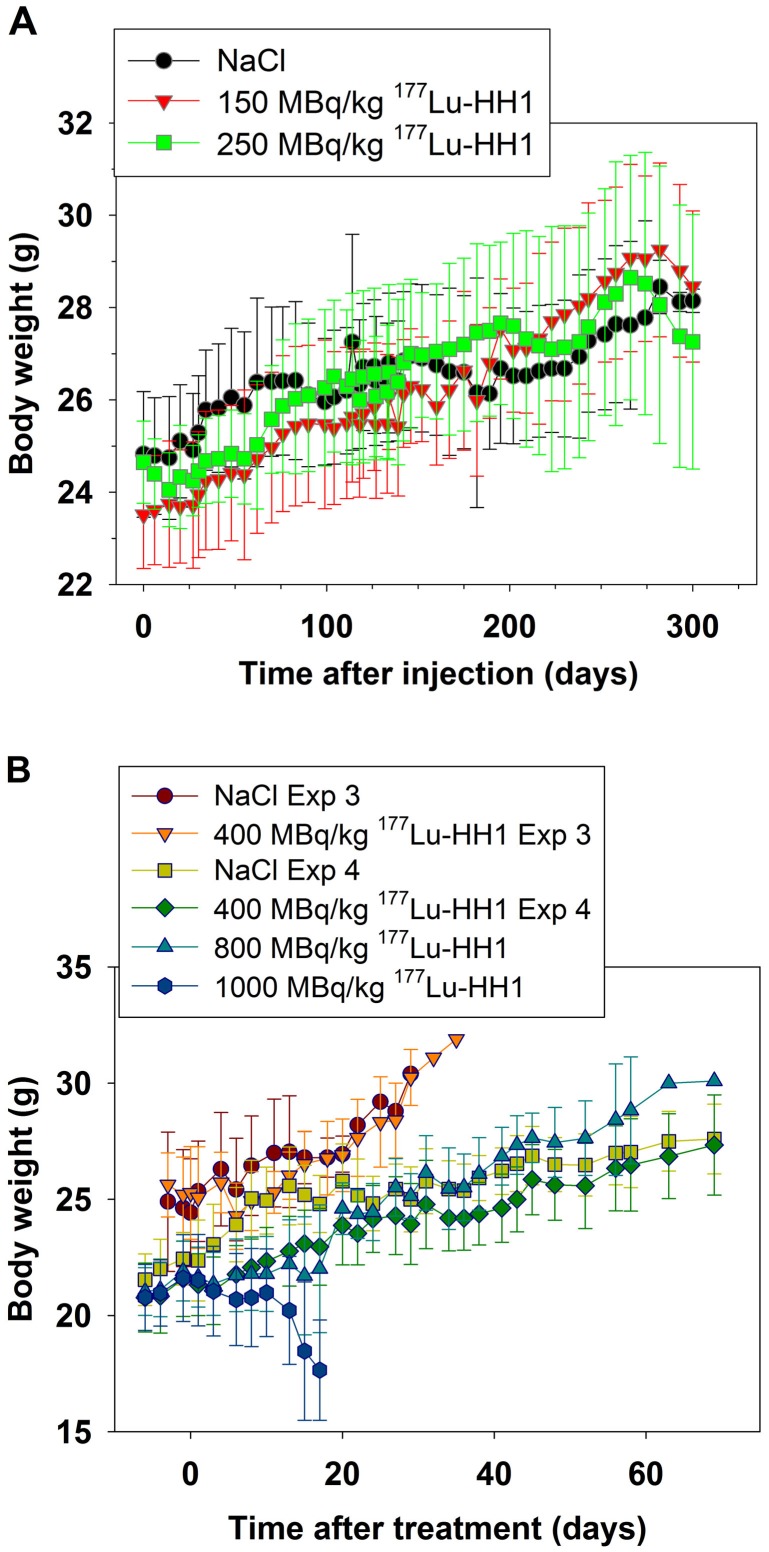
Effects of increasing dosages of ^177^Lu-HH1 on bodyweight of nude mice. Error bars  =  SD, N = 4–10. **A**. Mice without xenografts treated with saline, 150 or 250 MBq/kg ^177^Lu-HH1 (experiment 2). **B**. Mice with Ramos xenografts treated with saline or 400, 800 or 1000 MBq/kg ^177^Lu-HH1 (experiment 3 and 4).

There was no difference in average body weight for mice treated with saline, cold antibodies or 50–250 MBq/kg ^177^Lu-HH1 (Figure 3, data for cold antibodies and 50, 100 and 200 MBq/kg ^177^Lu-HH1 not shown). The mice treated with 400 MBq/kg ^177^Lu-HH1 had a lower average body weight than the mice treated with NaCl for the first 3 to 22 days after treatment injection, but the lower body weight was only significantly different in experiment 4 (p<0.04, Figure 3B).

For mice treated with 800 MBq/kg ^177^Lu-HH1 there was also a statistically significant decrease in body weight from day 3 to 17 after injection (p<0.03, Figure 3B). On days 16 and 17 after injection 4 of the mice showing radiation toxicity symptoms after treatment with 800 MBq/kg ^177^Lu-HH1 were euthanized. Hereafter, the average body weight increased similarly to that in the control group (p>0.1).

The average body weight of mice treated with 1000 MBq/kg ^177^Lu-HH1 decreased continuously from day 3 after treatment and was significantly lower than the body weight of mice treated with saline for all time points (p<0.03). The weight loss was severe after 10 days, which was considered to be an indirect symptom of radiation toxicity since no deleterious changes to the gastrointenstinal system were observed. The weight loss was probably related to less food intake of sick mice.

### Clinical Chemistry

**Figure 4:**
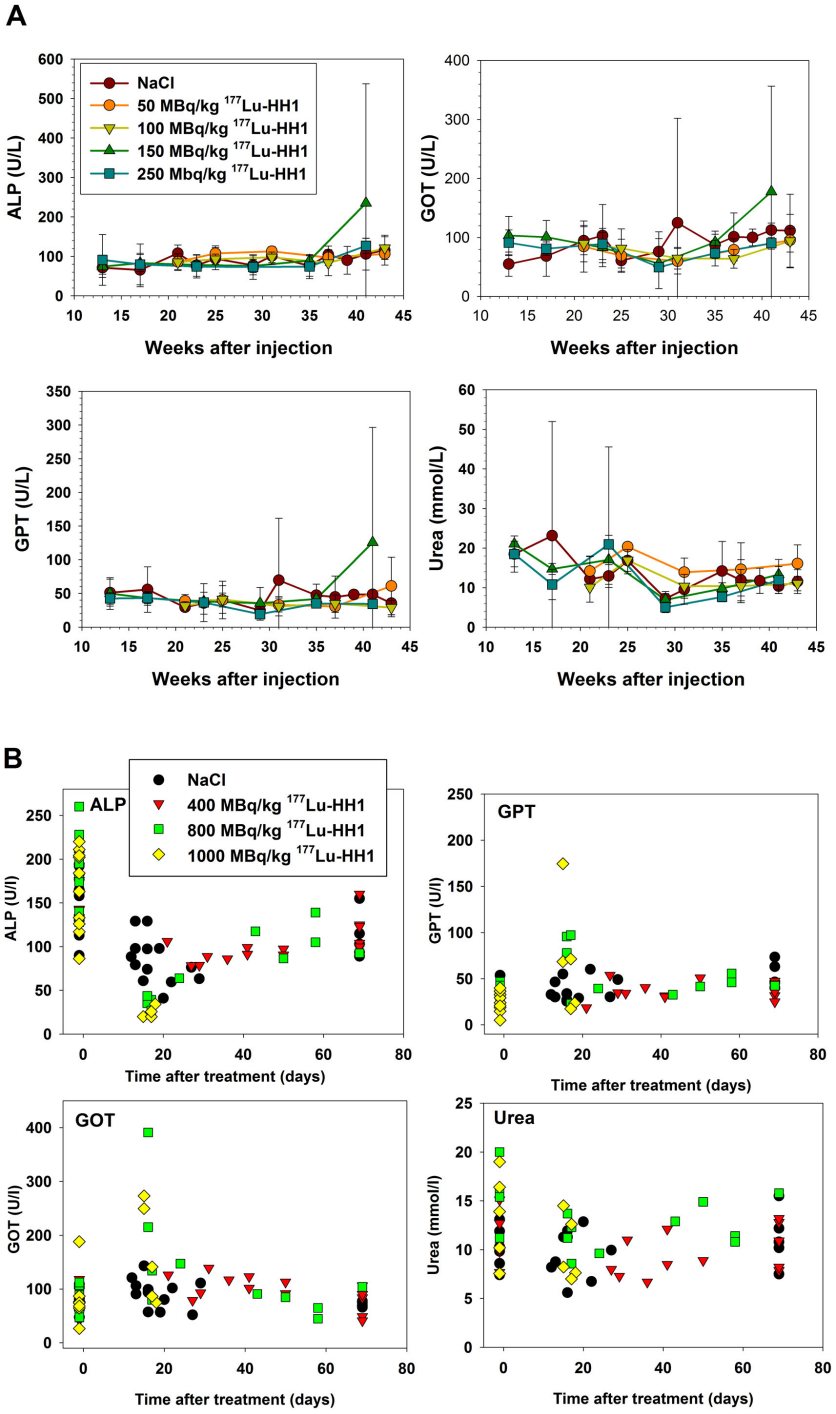
Effects of increasing dosage of ^177^Lu-HH1 on the levels of Alkaline phosphatase (ALP), Aspartate transaminase (GOT), Alanine transaminase (GPT) and Urea. **A**. Mice without xenografts treated with saline or 50, 100, 150 or 250 MBq/kg ^177^Lu-HH1 (experiments 1 and 2). Blood samples were taken every 3 to 4 weeks, N = 5–10. Error Bars: Standard deviation. **B**. Mice with Ramos xenografts treated with saline or 400, 800 or 1000 MBq/kg ^177^Lu-HH1 (experiment 3 and 4). Blood samples were taken before treatment and at euthanasia.

Treatment with dosages of ^177^Lu-HH1 between 50 and 400 MBq/kg, cold rituximab or HH1 did not significantly alter the average value of clinical chemistry parameters (Figure 4 A and B, data for treatments of 50 to 250 MBq/kg ^177^Lu-HH1 and cold rituximab and HH1 not shown).

For mice treated with 1000 MBq/kg ^177^Lu-HH1, significantly (p<0.05) lower average values of ALP, 24±6 U/L versus 92±28 U/L for NaCl, as well as significantly (p<0.05) higher average values of GOT; 165±92 U/L versus 85±29 U/L for NaCl, were observed at euthanasia (Figure 4B). There were no significant changes in the urea values. The same pattern was seen for the mice treated with 800 MBq/kg, but here the changes were not statistically significant. The mice treated with 800 or 1000 MBq/kg ^177^Lu-HH1 that had low values of ALP (below 50 U/l) and high values of GPT and GOT (above 80 U/l and 200 U/l, respectively) where the same animals that had to be euthanized due to symptoms of radiation toxicity (Figure 4B). The changes in the serum concentrations of these liver enzymes might indicate liver damage.

One mouse treated with 150 MBq/kg ^177^Lu-HH1 showed extreme values of ALP (774 U/L), GOT (496 U/L) and GPT (437 U/L) 41 weeks after injection of treatment. This explains the large error bars at this time point in Figure 4A. This mouse did not show any symptoms of illness or discomfort, hematology values were normal, and it was euthanized at the end of the experiment, not due to any clinical signs or symptoms. A mouse treated with NaCl (in experiment 1) showed extreme values of GOT (525 U/L) and GPT (257 U/L) 31 weeks after injection, and thus explains the large error bars in Figure 4 A at this time point. Hematology values were normal. The mouse was euthanized at this time point due to weigh loss and rectal prolapse.

### Histopathology

**Figure 5:**
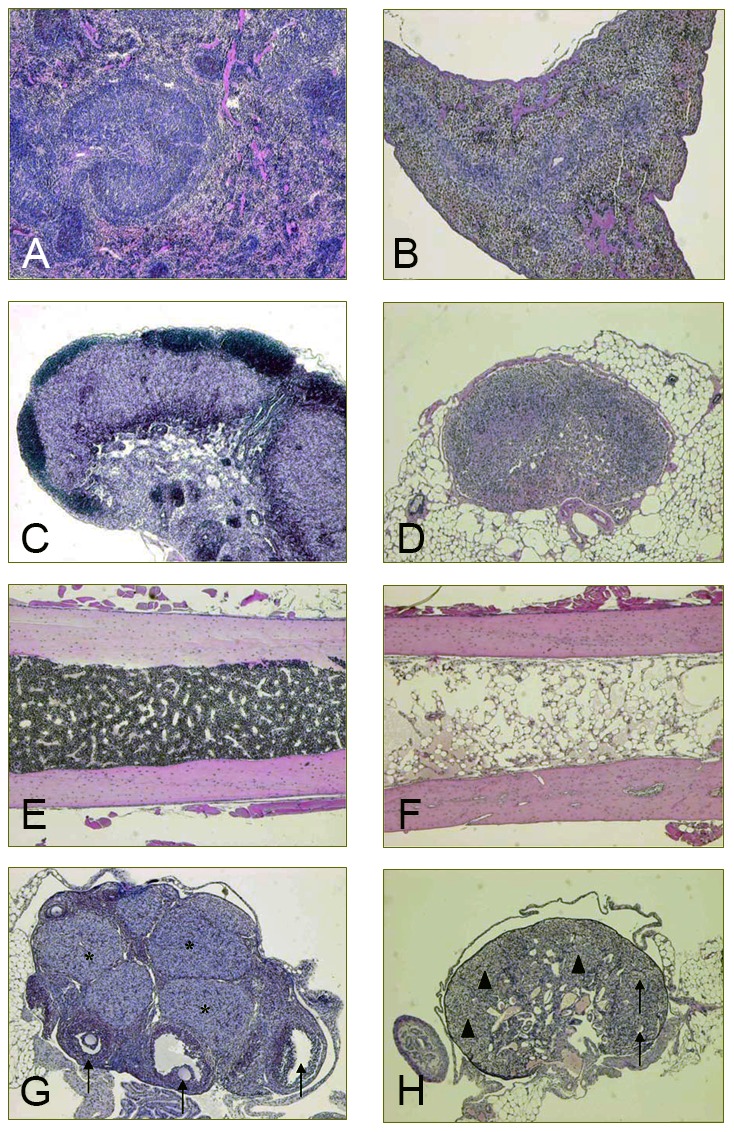
Representative pictures of main histological findings in mice treated with 1000/kg ^177^Lu-HH1 compared to control mice (H/E, 4X). **A**. Normal spleen from a control mouse. **B**. Severe lymphoid depletion and reduction of extramedullary hematopoiesis in the spleen from a mouse treated with 1000 MBq/kg. **C**. Normal lymph node from a control mouse showing diffuse interstitial cell hyperplasia. This is a common background finding noted in the lymph nodes from athymic nude mice. **D**. Severe lymphoid depletion noted in the lymph node in a mouse treated with 1000 MBq/kg. **E**. Normal bone marrow from a control mouse. **F**. Severe atrophy of the bone marrow in a mouse treated with 1000 MBq/kg. **G**. Ovary from a control mouse showing the presence of growing follicles (arrows) and corporea lutea (*). **H**. Ovary from a mouse treated with 1000 MBq/kg showing follicular atrophy (arrows) and severe hyperplasia of the interstitial cells (arrowheads).

The toxic effects were most evident and severe in tumor-bearing mice treated with 800 and 1000 MBq/kg ^177^Lu-HH1 (experiment 4). The main organs affected were the bone marrow, lymph nodes, spleen and ovaries (Table 2, Figure 5). Animals treated with 800 and 1000 MBq/kg of ^177^Lu-HH1 showed severe changes in the hemato-lymphopoietic system consisting of decrease in the incidence of extramedullary hematopoiesis in the spleen, lymphoid depletion in the spleen and lymph nodes, and atrophy of the bone marrow. The changes in the ovaries for the mice treated with 800 and 1000 MBq/kg ^177^Lu-HH1 were similar to those found in the mice treated with 50 to 250 MBq/kg ^177^Lu-HH1, but the severity was greater.

**Table 2:** Histology: summary of main findings.

Organ	Characteristic	% Incidence (No. mice with events/No. mice examined)		
**Exp 1–10 months end point**		**0.9% NaCl**	**50 MBq/kg**	**100 MBq/kg**
**Ovaries**	Atrophy	66.7 (2/3)	66.7 (4/6)	20 (1/5)
	Interstitial cell hyperplasia	66.7 (2/3)	100 (6/6)	100 (5/5)
**Exp. 2–6 months end point**		**0.9% NaCl**	**150 MBq/kg**	**250 MBq/kg**
**Ovaries**	Atrophy & interstitial cell hyperplasia	0 (0/2)	100 (4/4)	100 (1/1)
**Spleen**	Germinal center development	100 (2/2)	80 (4/5)	100 (3/3)
	Plasmacytosis	50 (1/2)	100 (5/5)	100 (3/3)
**Bone marrow**	Erythroid & Myeloid hyperplasia	0 (0/2)	0 (0/5)	100 (3/3)
**Exp. 2–10 months end point**		**0.9% NaCl**	**150 MBq/kg**	**250 MBq/kg**
**Ovaries**	Atrophy & interstitial cell hyperplasia	0 (0/4)	100 (5/5)	100 (3/3)
	Tubulostromal hyperplasia	0 (0/4)	80 (4/5)	33.3 (1/3)
**Exp 4.**		**0.9% NaCl**	**800 MBq/kg**	**1000 MBq/kg**
**Ovaries**	Atretic follicles	0 (0/10)	66.7 (6/9)	0 (0/2)
	Interstitial cell hyperplasia	0 (0/10)	44,4 (4/9)	0 (0/2)
**Spleen**	Lymphoid depletion	0 (0/10)	40 (4/10)	100 (6/6)
	Extramedullary hematopoiesis	90 (9/10)	50 (5/10)	0 (0/6)
**Bone Marrow**	Atrophy	0 (0/9)	44,4 (4/9)	100 (6/6)
**Lymph Nodes**	Lymphoid depletion	0 (0/10)	50 (5/10)	83,3 (5/6)
**Liver**	Hepatocellular vacuolation	0 (0/10)	10 (1/10)	66,7 (4/6)
	Hepatocellular necrosis	0 (0/10)	20 (2/10)	83,3 (5/6)
**Brain**	Hemorrhages	0 (0/10)	11,1 (1/9)	66,7 (4/6)
	Necrosis	0 (0/10)	14,3 (1/7)	50 (3/6)

The main changes observed in mice treated with 50 to 250 MBq/kg ^177^Lu-HH1 and euthanized after 6 and 10 months (experiments 1 and 2) were atrophy and interstitial cell hyperplasia in the ovaries and mild myeloid and erythroid hyperplasia in the bone marrow. In addition, a slightly increased incidence of plasmatocytosis and prominence of germinal centers was noted in the spleens of mice treated with 150 and 250 MBq/kg ^177^Lu-HH1. These changes were considered to be non-specific changes related to the increased susceptibility of nude mice to infections.

Mice treated with 50 and 100 MBq/kg did not have macroscopic tumors. However, the presence of microscopic lymphoma infiltration was observed in spleen, lymph nodes, pancreas or liver in both control and treated mice from experiment 1, with a slightly decreased incidence in treated animals. This change was characterized by the presence of dense sheets of round to polygonal cells up to 40 micrometers in diameter. The lymphoma infiltration was probably due to lymphoma cells from the subcutaneous xenografts with which these mice were originally implanted.

## Discussion

RIT with the residualizing and β-emitting ^177^Lu radioisotope targeted to the internalizing antigen CD37 by the HH1 antibody differs from the current non-internalizing CD20-directed RITs Bexxar and Zevalin. We have previously shown good therapeutic effects of ^177^Lu-HH1 in SCID mice [19] and relatively high tumor uptake and low normal tissue uptake in nude mice [21]. The expected target organ for toxicity of ^177^Lu-HH1 is bone marrow due to retention of the RIC in the blood and, binding to tumor cells if present, in the bone marrow. The toxicity is expected to be transient since normal stem cells do not express CD37 and will therefore be damaged only by cross-fire radiation and not by direct radiation from bound RIC.

In the present study, we have demonstrated that the maximum tolerated dosage (MTD) of ^177^Lu-HH1 in nude mice is between 400 and 800 MBq/kg. The study indicates that the dose limiting organ is the bone marrow. This finding is in agreement with previous studies showing that the most common and dose-limiting side effect of RIT in lymphoma is bone marrow toxicity [24]. In addition, both the MTD and the dose-limiting organ observations are in good agreement with results from another study of ^177^Lu-CC49 (a murine IgG_1_ reactive with the tumor-associated antigen TAG-72) [25]. This study reported that 55% of the nude mice bearing subcutaneous human colon carcinomas treated with 500 µCi (equivalent to 740 MBq/kg for a 25 g mouse) of ^177^Lu-CC49 suffered toxic death related to bone marrow toxicity. The MTD of ^177^Lu-HH1 found in nude mice in this study was approximately 10 times higher than the MTD found for SCID mice, which was between 50 and 100 MBq/kg [22]. The MTD in SCID mice of a ^177^Lu-labeled anti-CD74 antibody was reported to be 72 MBq/kg [26]. It is, therefore, apparent that ^177^Lu-labeled antibodies are more toxic for SCID mice than for nude mice. This was expected due to the impaired DNA-damage-repair capacity of the SCID model [20].

A phase I clinical study of ^177^Lu-rituximab resulted in a MTD of 47 MBq/kg [27] and a phase I clinical study of ^177^Lu-J591 (a mAb that targets the anti–prostate-specific membrane antigen) resulted in a MTD of 74 MBq/kg [28], indicating that nude mice can tolerate much higher doses than humans. The reason why the MTD for humans is more similar to the MTD for SCID mice than for nude mice may be related to differences in DNA repair capacity between rodent and human cells, or it could be attributed to the large differences in tumor targeting and normal tissue uptake and retention between rodents and humans. HH1 is not cross reactive to mouse B-cells and hence, only non-specific binding of the RIC and cross-radiation from specifically bound RIC to the tumor xenograft will contribute to the absorbed radiation dose in nude mice.

Ovarian atrophy and interstitial cell hyperplasia were observed in some of the treated animals. The interstitial cell hyperplasia has been reported to accompany ovarian atrophic changes [29], [30]. The present study does not allow to conclude if these changes are age-related or if there is any contribution from the ^177^Lu-HH1 treatment. We hypothesize that the increase in ovary atrophy observed in the treated animals could be related to the radiation dose to ovaries due to cross-fire radiation from the kidneys, where ^177^Lu-HH1 has a modest uptake [21]. The ovaries are separated from the kidneys by a thin layer of fat so that part of the beta-radiation from ^177^Lu in the kidneys can reach the ovaries, since the maximum range of the beta-radiation is 1.6 mm. If this is the cause of the changes observed in mouse ovaries, this effect should not be expected in humans.

In summary, the current study of ^177^Lu-HH1 did confirm that the RIC has low toxicity in non-target tissues of nude mice for dosages lower than 400 MBq/kg and moderate toxicity at 400 MBq/kg. The dose-limiting toxicity occurred in the bone marrow and manifested as low blood cell counts a few weeks after administration of the RIC. The long term follow up data indicated only modest late toxicity in this murine model of human B-cell NHL. A dose to tumor of 23 Gy could be achieved by treating with 400 MBq/kg according to biodistribution and dosimetry studies in nude mice with Daudi xenografts [21] and should be sufficient to achieve complete remission, if delivered relatively uniformly, to a macroscopic NHL-tumor.

The toxicity profile of ^177^Lu-HH1 presented in this paper, combined with the previously published study demonstrate that ^177^Lu-HH1 has a similar biodistribution in nude mice to that of the clinically tested ^177^Lu-rituximab [21], [27]. This study suggests that ^177^Lu-HH1 is suitable for clinical evaluations.

## Conclusion

^177^Lu-DOTA-HH1 was well tolerated at dosages well above those considered relevant for RIT in patients with B-cell derived malignancies. The MTD of ^177^Lu-HH1 in nude mice was between 400 and 800 MBq/kg. ^177^Lu-HH1 showed moderate toxicity in non-target tissues of nude mice for dosages lower than 400 MBq/kg. The dose-limiting toxicity occurred in the bone marrow and manifested itself as low blood cell counts a few weeks after administration of the RIC followed by a return to normal ranges. The long term follow up data indicated only modest late toxicity in this murine model of human B-cell NHL.
